# Correction: Chemical transformation of the multibudding yeast, *Aureobasidium pullulans*

**DOI:** 10.1083/jcb.20240211404042025c

**Published:** 2025-04-10

**Authors:** Alison C.E. Wirshing, Claudia A. Petrucco, Daniel J. Lew

Vol. 223, No. 10 | https://doi.org/10.1083/jcb.202402114 | June 27, 2024

The authors regret that, in their original article, “100 mM LiOAc” was missing from the Competence Buffer mentioned in the Materials and methods section, in the first paragraph of the section named “*A. pullulans* transformation.” The corrected text is shown below.

This error appears in print and in PDFs downloaded on or before April 7, 2025.

## *A. pullulans* transformation

To make competent cells, a single *A. pullulans* colony was inoculated into 5 ml of YPD (4% glucose) and grown overnight (∼18 h) at 24°C with agitation. The following day, the overnight culture was diluted to a final density of ∼5 × 10^6^ cells/ml in 50 ml of YPD (4% glucose) in a 250-ml flask. Cells were grown at 22–24°C with agitation at 200 rpm for ∼2–3 h until reaching a density of ∼10^7^ cells/ml, unless indicated otherwise. Cells were harvested via centrifugation at 2,254 rcf for 8 min in 50 ml conical tubes and then resuspended in 1 ml sterile room-temperature deionized water and transferred to a 1.5 ml Eppendorf tube before pelleting at 9,391 rcf for 10 s. The cells were rinsed once in 500-µl sterile Competence Buffer (10 mM Tris pH 8, 1 mM EDTA, **100 mM LiOAc**, 1 M sorbitol) and pelleted at 9,391 rcf for 10 s. Finally, cells were resuspended at a final density of ∼2 × 10^9^ cells/ml in Competence Buffer, usually ∼250 µl, and divided into aliquots in 1.5 ml Eppendorf tubes with ∼10^8^ cells in each aliquot (∼60–65 µl in each). For conditions with amino acids supplemented into the Competence Buffer, Complete Supplement Mixture minus uracil (1004-100; Sunrise Science Products) and uracil (600-621-9; Sigma-Aldrich) were added to the Competence Buffer at a final concentration of 0.96 and 0.025 g/l, respectively. This corresponds to an amino acid concentration 1.25× the concentration used in a standard complete synthetic medium previously shown to improve the transformation of *S. cerevisiae* (Yu et al., 2016). Competence Buffer with amino acids was prepared fresh just before use and filter-sterilized. To make frozen competent cells for future use, aliquots of cells in Competence Buffer were transferred to a −80°C freezer.

